# *Akkermansia Muciniphila* Potentiates the Antitumor Efficacy of FOLFOX in Colon Cancer

**DOI:** 10.3389/fphar.2021.725583

**Published:** 2021-09-17

**Authors:** Xiaoying Hou, Pei Zhang, Hongzhi Du, Weihua Chu, Ruiqi Sun, Siyuan Qin, Yuan Tian, Zunjian Zhang, Fengguo Xu

**Affiliations:** ^1^ Key Laboratory of Drug Quality Control and Pharmacovigilance (Ministry of Education), State Key Laboratory of Natural Medicine, China Pharmaceutical University, Nanjing, China; ^2^ School of Pharmacy, Hubei University of Chinese Medicine, Wuhan, China; ^3^ School of Life Science and Technology, China Pharmaceutical University, Nanjing, China

**Keywords:** FOLFOX, oxaliplatin, colon cancer, Akkermansia muciniphila, pharmacomicrobiomics, metabolomics

## Abstract

FOLFOX (oxaliplatin, fluorouracil and calcium folinate) is the first-line chemotherapy regimen for colon cancer therapy in the clinic. It provides superior efficacy than oxaliplatin alone, but the underlying mechanism remains unclear. In the present study, pharmacomicrobiomics integrated with metabolomics was conducted to uncover the role of the gut microbiome behind this. First, *in vivo* study demonstrated that FOLFOX exhibited better efficacy than oxaliplatin alone in colon cancer animal models. Second, 16S rDNA gene sequencing analysis showed that the abundance of *Akkermansia muciniphila* (*A. muciniphila*) remarkably increased in the FOLFOX treated individuals and positively correlated with the therapeutic effect. Third, further exploration confirmed *A. muciniphila* colonization significantly enhanced the anti-cancer efficacy of FOLFOX. Last, metabolomics analysis suggested dipeptides containing branched-chain amino acid (BCAA) might be responsible for gut bacteria mediated FOLFOX efficacy. In conclusion, our study revealed the key role of *A. muciniphila* in mediating FOLFOX efficacy, and manipulating *A. muciniphila* might serve as a novel strategy for colon cancer therapy.

**Graphical Abstract:**
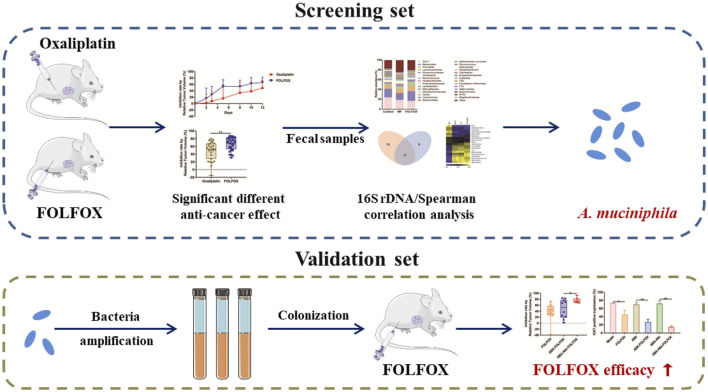


## Introduction

Oxaliplatin is a diamine cyclohexane platinum derivative that shows better tolerance than cisplatin in terms of nephrotoxicity (Yuan et al., 2020). Like other platinum compounds, oxaliplatin acts primarily through binding with inter- and intra-strand cross-links in DNA, forming DNA adducts, and thereby inhibiting cell DNA synthesis (Zimmermann et al., 2020). However, oxaliplatin reached only ∼10% response rate when applied alone in clinical practice. Meanwhile, severe peripheral sensory neuropathy occurs in ∼10% of patients after six treatment cycles and the rate reaches ∼50% after nine cycles, which largely limited its further application (Cvitkovic and Bekradda, 1999; Zhang et al., 2020). Therefore, oxaliplatin is often applied in combination with fluorouracil (5-FU) and calcium folinate in the clinic (i.e., FOLFOX). According to the latest version of the National Comprehensive Cancer Network (NCCN) guidelines (https://www.nccn.org/), FOLFOX is the first-line chemotherapy regimen for advanced colon cancer (Abraham et al., 2020). Compared to oxaliplatin alone, FOLFOX achieves prominently increased efficacy and attenuated toxicity. Recent studies have suggested that gut microbiota, immune regulation, and tyrosine kinase Src might influence the anti-cancer effect of FOLFOX (Parseghian et al., 2017; Dosset et al., 2018; Chang et al., 2020). However, the underlying mechanism for the increased efficacy of FOLFOX remains unclear (Machover et al., 1996).

An increasing number of studies suggested that the intrinsic gut microbiome is one of the important factors affecting the efficacy of chemotherapeutics (Liu et al., 2020; Zhang et al., 2020). Pharmacomicrobiomics is an emerging field that investigates the interplay of microbiome variation and drug response and disposition (Doestzada et al., 2018). On the other hand, as the significant role of intestinal flora in mediating drug efficacy is gradually recognized, the application of microflora transplantation alone or in combination with other drugs has achieved surprisingly satisfactory outcomes in the clinic. For example, fecal microbiota transplantation was used in the treatment of pseudomembranous colitis and sepsis, which could overcome traditional drug resistance and reach distinguished therapeutic efficacy (Gupta and Khanna, 2017; Panigrahi et al., 2017). In the case of FOLFOX, we speculated that intestinal flora might also play key roles in mediating the superior efficacy.

In the current study, pharmacomicrobiomics and metabolomics were applied to investigate the involvement of gut microbiota in the efficacy of FOLFOX. First, a colon cancer xenograft model was established to compare the efficacy of oxaliplatin and FOLFOX. Then, 16S rDNA gene sequencing analysis and correlation analysis were conducted to screen differential gut microbiota after oxaliplatin and FOLFOX treatments. Further, bacterial colonization combined with FOLFOX was performed to verify the influence of the focused bacteria on the therapeutic effect. Finally, metabolomics analysis was conducted to discover metabolites derived from gut microbiota.

## Materials and Methods

### Chemicals and Reagents

Oxaliplatin and Calcium Folinate Injection were both obtained from Aosaikang (Jiangsu, China), and 5-FU Injection was obtained from SunRise (Shanghai, China). Oxaliplatin and FOLFOX Injection were prepared according to clinical guidelines as well as existing studies (Iida et al., 2013; Yang et al., 2016). Chemicals including O-Methoxyamine hydrochloride, N-methyl-N-trifluoroacetamide (MSTFA) and cortisone acetate were purchased from Sigma–Aldrich (St.Louis, MO, United States). Vancomycin (MB1260), Ampicillin (MB1507), Neomycin sulfate (MB1716) and Metronidazole (MB2200) were purchased from Meilunbio (Dalian, China).

### Colon Cancer Cell

Mice colon cancer cell line CT-26 was purchased from the Cell Bank of the Institute of Biochemistry and Cell Biology, Chinese Academy of Sciences (Shanghai, China). The cells were cultured in RPMI-1640 (Gibco, Grand Island, United States) supplied with 10% Fetal Bovine Serum (Gibco, Grand Island, United States) in a humidified atmosphere with 5% CO_2_ at 37°C.

### Colon Cancer Xenograft Model Construction and Sample Collection

Five to six-week-old male BALB/c mice were provided by Beijing Vital River Laboratory Animal Technology Co. Ltd. (License No. SCXK 2019-0001). The mice were housed in a temperature-controlled environment (24 ± 2°C) under a 12/12 h-dark/light cycle. The study was conducted in accordance with the Guide for the Care and Use of Laboratory Animals and approved by the Animals Ethics Committee of China Pharmaceutical University (License No: SYXK 2018-0019).

After acclimation for 1 week, approximately 1×10^6^ CT-26 cells were injected subcutaneously into the flank of mice. When the tumors reached to about 100 mm^3^, mice were randomly allocated to one of the groups (day 0), i.e., model for oxaliplatin (MO, *n* = 8), oxaliplatin treatment (Oxaliplatin, *n* = 27), model for FOLFOX (MF, *n* = 6), FOLFOX treatment (FOLFOX, *n* = 33). Based on previous studies, oxaliplatin (10 mg/kg) was intraperitoneally administrated twice a week (Yang et al., 2016), and FOLFOX (oxaliplatin 6mg/kg, 2 h after 5-FU 50 mg/kg and Calcium Folinate 90m g/kg treatment) was intraperitoneally administrated once a week (Robinson et al., 2013; Limani et al., 2016). MO and MF individuals were treated with corresponding vehicles. Individuals in the control group (*n* = 7) received neither tumor cell injection nor drug treatment. Tumor volume was monitored by a Vernier Caliper throughout the whole experimental period. All the mice were sacrificed at the end of the experiment (day 12), fecal samples were collected for 16S rDNA gene sequencing analysis and non-target metabolomics analysis.

Tumor volume (TV), Relative tumor volume (RTV) and inhibition rate were calculated by the following formulas:

$$\mathrm{TV}\left(mm_{3}\right)=\frac{A}{2}\times B^{2}\text{,}$$

where A represents the longest diameter of tumor, and B represents the shortest diameter;

$$\mathrm{RTV =}\frac{V_{t}}{V_{0}},$$

where V_0_ represents the tumor volume of day 0 (the day of first oxaliplatin administration), V_t_ represents the tumor volume of day t; Inhibition rate by Relative Tumor Volume (%) = (1−RTV_t_ /RTV_m_) × 100%, where RTV_m_ represents the RTV of model group, and RTV_t_ represents the RTV of treatment group.

### Bacterial DNA Extraction and Quantification

Total bacterial DNA was isolated from fecal with Stool Genomic DNA Kit (CWBIO, Beijing, China) according to the manufacturer’s instructions. DNA quantification was conducted by a NanoDrop 2,000 (Thermo Fisher Scientific, Waltham, United States).

### 16S rDNA Gene Sequencing Analysis

The DNA integrity was checked by 1% agarose gels electrophoresis. PCR amplification was performed spanning the V3-V4 hypervariable regions of the bacterial 16S ribosomal RNA gene with the conventional barcoded universal bacterial primers 338F (5′-ACT​CCT​ACG​GGA​GGC​AGC​AG-3′) and 806R (5′-GGACTACHVGGGTWTCTAAT-3′), and sequenced with an Illumina Hiseq 2500 platform (Illumina, San Diego, United States) (Holm et al., 2019; Wan et al., 2019; Chen X. et al., 2020). Raw fastq files were filtered by the Quantitative Insights in the Microbial Ecology software. High-quality sequences were clustered into Operational Taxonomic Units (OTUs) with the similarity threshold of 97% by USEARCH UPARSE (Feng et al., 2019). Then, OTUs were classified into kingdom, phylum, class, order, family, and genus levels referring to the Greengenes database (Shikany et al., 2019), and eventually an OTU table was created. The parameter α-Diversity (Chao1/Shannon/Simpson) was used to reflect the bacterial gene diversity. Wilcoxon rank-sum test was applied to identify differential taxa between groups (taxa with *p* < 0.1 was screened). Bacteria that existed in less than 50% samples were excluded (Feng et al., 2019). Phylogenetic Investigation of Communities by Reconstruction of Unobserved States (PICRUSt) was applied to predict the potential function of microbial communities based on the Kyoto Encyclopedia of Genes and Genomes (KEGG) pathway database as previously reported (Park et al., 2020). Data concerning the samples included in this study are deposited in the NCBI BioProject database under BioProject accession number PRJNA706146.

### Bacteria Culture

*Akkermansia muciniphila* (*A. muciniphila*, ATCC BAA-835) was purchased from American Type Culture Collection (Manassas, VA, United States), the bacterium was cultured in Brain Heart Infusion Agar at 37°C under anaerobic condition.

### Animal Experiment Evaluating *A. muciniphila* on FOLFOX Efficacy

A broad-spectrum antibiotics mixture namely ABX consisting of Vancomycin (100 mg/kg), Neomycin sulfate (200 mg/kg), Metronidazole (200 mg/kg) and Ampicillin (200 mg/kg) was intragastrically administrated to mice every day for five consecutive days (day −14 to −9) to deplete gut microbiota and decrease its α-Diversity (Gong et al., 2019). The mice were then treated with *A. muciniphila* by gavage at 1 × 10^8^ colony forming unit (cfu)/mouse every other day until the end of the experiment (day −7 to 12). Fecal samples were collected at day −7 and 0 for transplantation efficiency verification. CT-26 cells (approximately 1 × 10^6^) were injected subcutaneously into the flank of mice at day −7. When the tumor volumes reached about 100 mm^3^, mice were randomly allocated to one of the following groups and the day was marked as day 0: Model (*n* = 10), FOLFOX (*n* = 10), ABX (*n* = 10), ABX-FOLFOX (*n* = 10), ABX-Akk (*n* = 10), ABX-Akk-FOLFOX (*n* = 10). FOLFOX was administrated once a week (day 2 and 9). Tumor volume was monitored by a Vernier Caliper throughout the experiment. All the mice were sacrificed at the end of the experiment (day 12), tumors were removed and prepared for immunohistochemistry analysis.

### Quantitative Polymerase Chain Reaction

Relative levels of *A. muciniphila* were quantified by qPCR (Tsoi et al., 2017). Total bacterial genome DNA isolation and quantification were conducted as mentioned above. Then, qPCR was performed using SYBR Green Ι Master (Roche Diagnostics, Basel, Switzerland) on a LightCycler 480 instrument (Roche) following the manufacturer’s instructions. The levels of *A. muciniphila* were calculated according to the 2^–ΔΔCT^ method (Wang et al., 2021). Information on PCR primers was provided in Supplementary Table S1.

### Histopathology

Tumors were fixed in formalin and embedded in paraffin. Sections were then subjected for hematoxylin and eosin (HE) staining and Ki67 immunohistochemistry detection as previously reported (Hou et al., 2018).

### Non-Target Metabolomics Analysis

Methods for fecal sample non-target metabolomics analysis were presented in our previous studies (Zhang et al., 2017a; Gao et al., 2019). Briefly, gas chromatography-mass spectrometry (GC-MS) analysis was performed on a GC-MS-QP2010 Ultra (Shimadzu Inc., Kyoto, Japan) with an Rtx-5MS capillary column (30.0 m × 0.25 mm × 0.25 μm). Liquid chromatography-mass spectrometry (LC-MS) detection was carried out on an ultra-flow liquid chromatography system coupled with ion trap/time-off light hybrid mass spectrometry (UFLC-IT/TOF-MS, Shimadzu Inc., Kyoto, Japan) and compounds were separated by a Phenomenex Kinetex C18 column (100 × 2.1 mm, 2.6 μm). Details on sample preparation, instrument parameters, metabolite annotation, quality control, and data analysis were provided in the supporting information.

### Statistical Analysis

Spearman’s correlation analysis was applied to test the correlation between fecal bacterial abundance and pharmacodynamic indices (IR, RTV, TV, and body weight) or metabolite intensities. Data analysis and graph plotting were performed by GraphPad Prism 8 software (GraphPad Software Inc., La Jolla, CA, United States). The results were presented as mean ± SD. Independent unpaired two-tailed Student’s *t* test was performed to evaluate the differences between two groups, unless elsewhere specified.

## Results

### FOLFOX Exhibited Superior Therapeutic Effect Than Oxaliplatin

**FIGURE 1:**
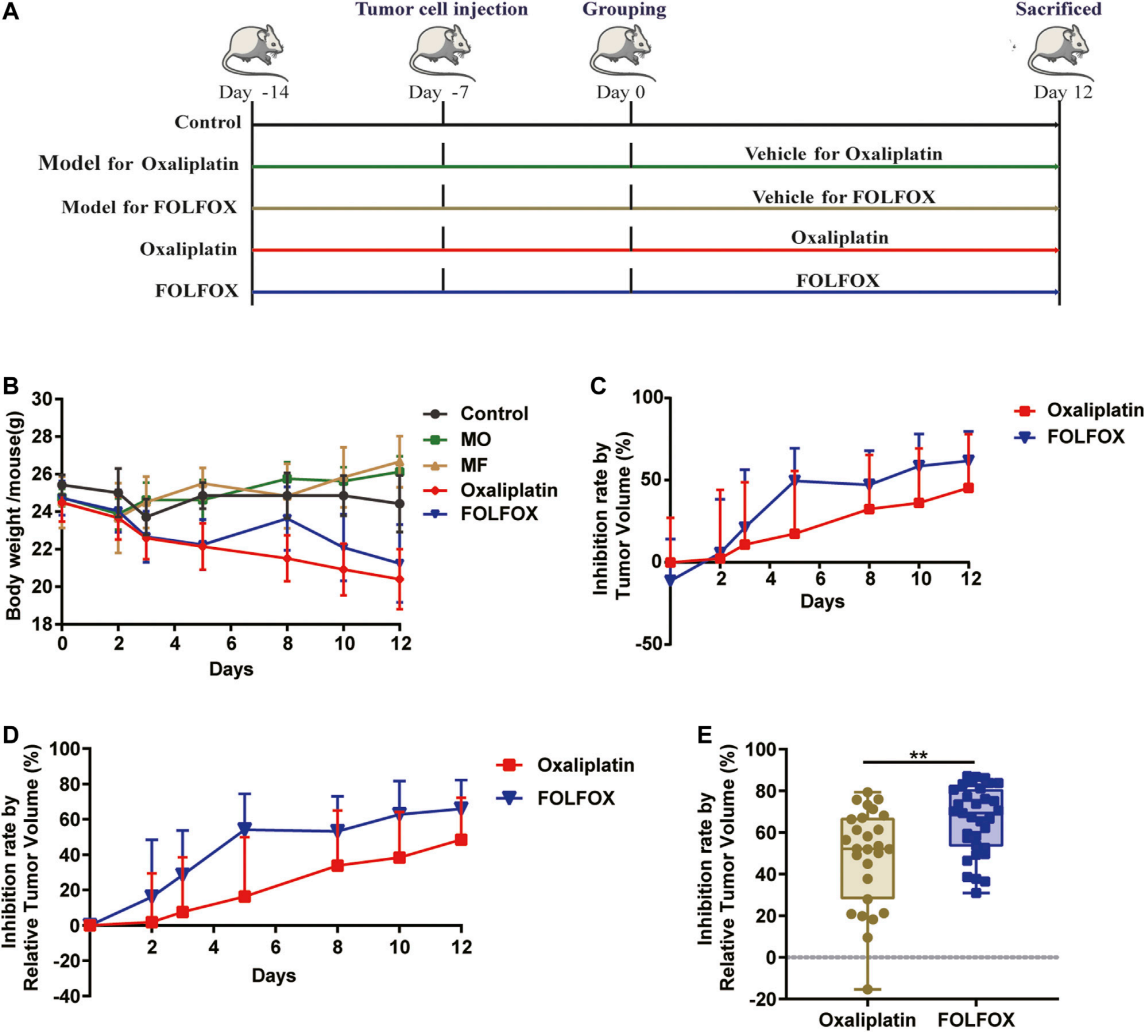
FOLFOX exhibited better chemotherapeutic efficacy in colon cancer xenograft model than oxaliplatin alone. **(A)** Schematic of the pharmacodynamic evaluation of oxaliplatin and FOLFOX in CT-26 colon cancer xenograft model. **(B)** Influence of oxaliplatin and FOLFOX on the body weight of tumor bearing mice. Inhibition rate was calculated by **(C)** TV and **(D)** RTV. **(E)** Inhibition rate calculated by RTV at the end of the experiment (day 12). Data were presented as mean ± SD. The *p*-values < 0.05 were considered statistically significant, *p <0.05, **p < 0.01.

In this study, a colon cancer xenograft model was constructed to evaluate the anti-cancer effect of oxaliplatin and FOLFOX (Figure 1A). Consistent with clinical practice and existing literature (Cvitkovic and Bekradda, 1999; Hoff et al., 2012), oxaliplatin caused more severe adverse effects than FOLFOX, manifested by a larger body weight reduction (Figure 1B). Moreover, the tumor development was more significantly inhibited with FOLFOX treatment compared to oxaliplatin throughout the whole experiment (Figures 1C,D). At the end of the experiment (day 12), significant difference in tumor inhibition rate (IR) (*p* < 0.01) was observed between the FOLFOX and oxaliplatin treated individuals (Figure 1E). Taken together, FOLFOX exhibited a superior therapeutic effect than oxaliplatin alone in colon cancer xenograft mice.

### Oxaliplatin and FOLFOX Showed Different Influence on the Gut Microbiota

Many studies have illustrated important roles of gut microbiota in mediating chemotherapy efficacy (Roy and Trinchieri, 2017), primarily through affecting drug biotransformation directly and interacting with the host indirectly (Vivarelli et al., 2019; Zimmermann et al., 2019). To investigate the underlying mechanism for the superior therapeutic effect of FOLFOX, fecal samples were collected at the end of the experiment and all the samples were subjected to 16S rDNA gene sequencing analysis.

**FIGURE 2:**
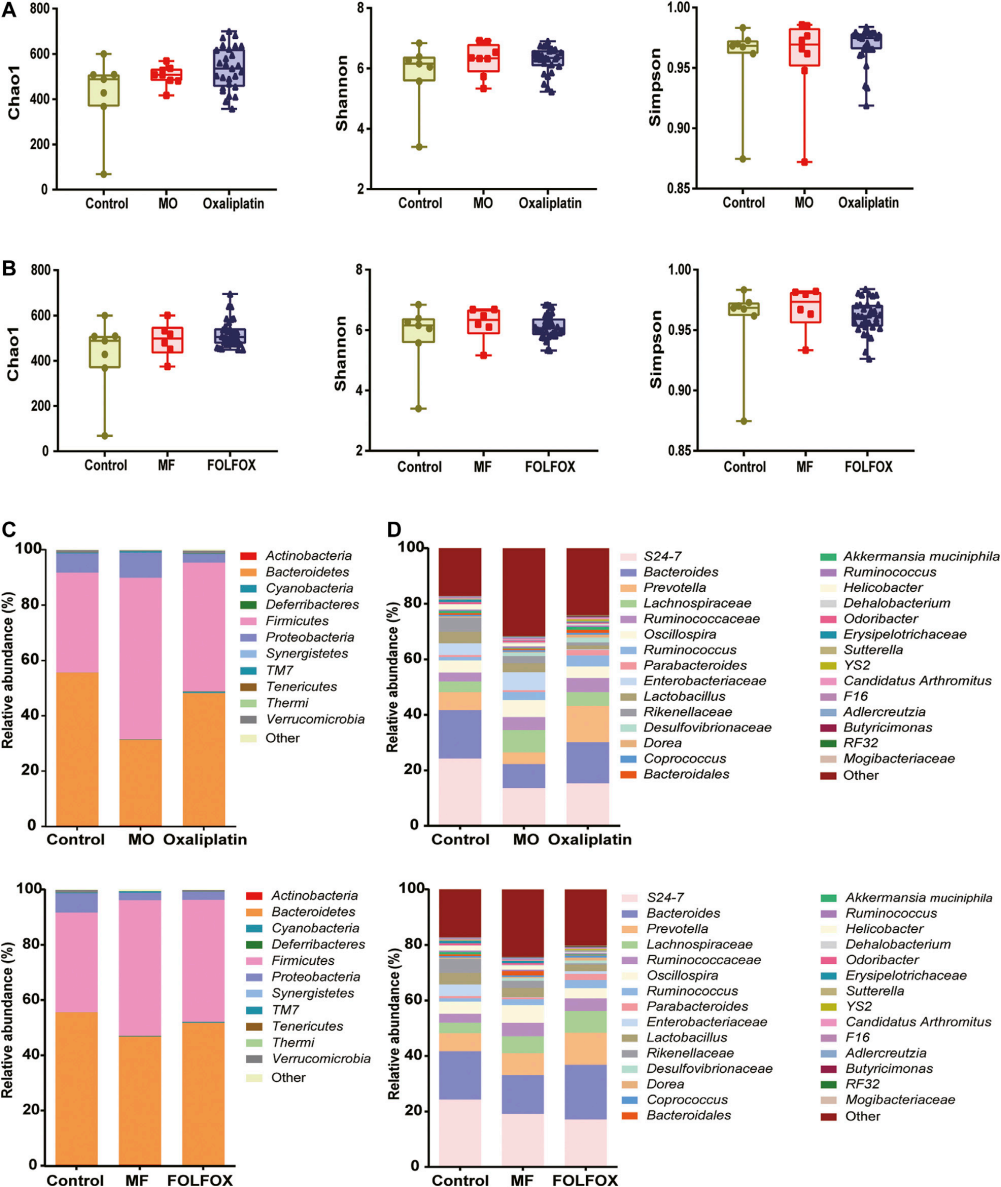
Oxaliplatin and FOLFOX could reverse the disordered distribution of gut microbiota induced by tumor. **(A, B)** Change of α-Diversity estimated by Chao 1, Shannon and Simpson estimator. Taxonomic distributions of bacteria based on fecal 16S rDNA gene sequencing data at **(C)** phylum and **(D)** genus level after oxaliplatin or FOLFOX treatment.

As is shown in Figures 2A,B, there was no significant difference in the α-Diversity for the model, FOLFOX, or oxaliplatin individuals compared to the controls indicated by Chao1, Shannon, and Simpson. This suggested that no dramatic changes in the overall microbial community richness were induced by the tumor model construction or the anti-cancer treatment. Notably, both FOLFOX, and oxaliplatin could reverse the disorder of gut microbiota induced by tumor at phylum, class, order, family, or genus level (Figures 2C,D and Supplementary Figure S1). At phylum level (Figure 2C), the control group was dominated by *Bacteroidetes* (55.27%), and *Firmicutes* represented the dominant bacteria in the MO (Model for oxaliplatin, 58.36%) and MF (Model for FOLFOX, 49.09%) groups, while the abundance of *Bacteroidetes* was reversed in the Oxaliplatin (48.00%) and FOLFOX (51.44%) groups. At the genus level, 21 significantly changed bacterial genera were obtained from MO *vs.* Oxaliplatin group (Supplementary Figure S2), and 19 bacterial genera from MF *vs.* FOLFOX group (Supplementary Figure S3). Moreover, according to LEfSe analysis based on KEGG pathways, oxaliplatin could increase the carbohydrate and nucleotide metabolism of gut microbiota (Supplementary Figure S4A), while carbohydrate and lipid metabolism was elevated in the FOLFOX group (Supplementary Figure S4B) (LDA score > 2.0 with *p* < 0.05). Taken together, the 16S rDNA gene sequencing analysis suggested an altered gut microbiota composition and function after oxaliplatin and FOLFOX treatment, implicating potential roles of the gut bacterial community in chemotherapy outcome.

**FIGURE 3:**
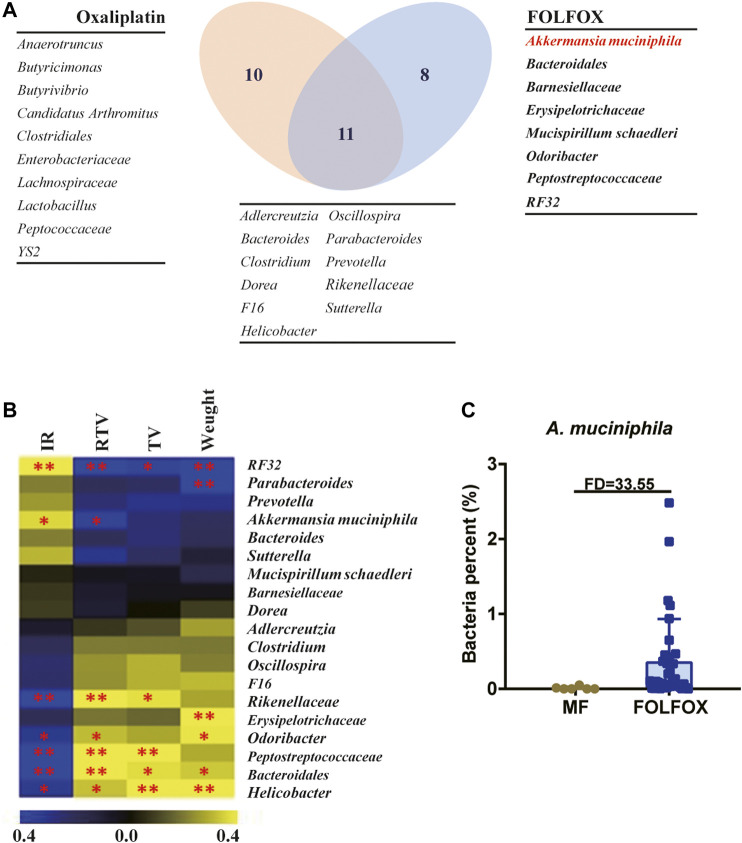
*A. muciniphila* might be the key bacteria accounting for the superior efficacy of FOLFOX. **(A)** Venn diagram illustrating the shared/unique differential gut microbiota OTUs after oxaliplatin and FOLFOX treatment. **(B)** Heatmap of Spearman correlation coefficient between pharmacodynamic indices after FOLFOX treatment and abundance of changed bacterial genera. The intensity of the colors represents the degree of association between the level of pharmacodynamic indices and abundance of changed bacterial genera determined by Spearman’s correlations. IR, Inhibition rate by Relative Tumor Volume. **(C)** Relative abundance of *A. muciniphila* in the MF and FOLFOX groups. The *p*-values < 0.05 were considered statistically significant, *p < 0.05, **p < 0.01.

Here, changes in the distribution and function of gut microbiota in tumor-bearing mice were observed after oxaliplatin and FOLFOX treatment, but the actual role of gut bacteria in the improved efficacy of FOLFOX requires further exploration. For this purpose, we listed the significantly changed bacteria from the comparisons of MO *vs.* Oxaliplatin and MF *vs.* FOLFOX, respectively. As is shown in the Venn diagram (Figure 3A), in total there were 29 bacterial genera, among which 11 were shared by the both treatments, 10 and 8 were unique to oxaliplatin and FOLFOX treatment, respectively. Meanwhile, Spearman correlation analysis was performed to reveal the correlation between differential bacteria and chemotherapeutic efficacy (IR, RTV, TV, and Weight) after FOLFOX treatment (Figure 3B and Supplementary Figure S5). The abundance of *RF32* and *A. muciniphila* was positively correlated with the IR of FOLFOX (*p* < 0.05). According to existing literature, *A. muciniphila* is known to associate with the improved prognosis of cancer patients and shows beneficial effects on metabolic disorders as well (Everard et al., 2013; Routy et al., 2018). Considering *A. muciniphila* had the highest fold change (33.55) between the MF and FOLFOX groups among all the bacteria (Figure 3C and Supplementary Figure S3), we conducted further experiments to validate its function.

### *A. Muciniphila* Colonization Significantly Increased FOLFOX Efficacy

**FIGURE 4:**
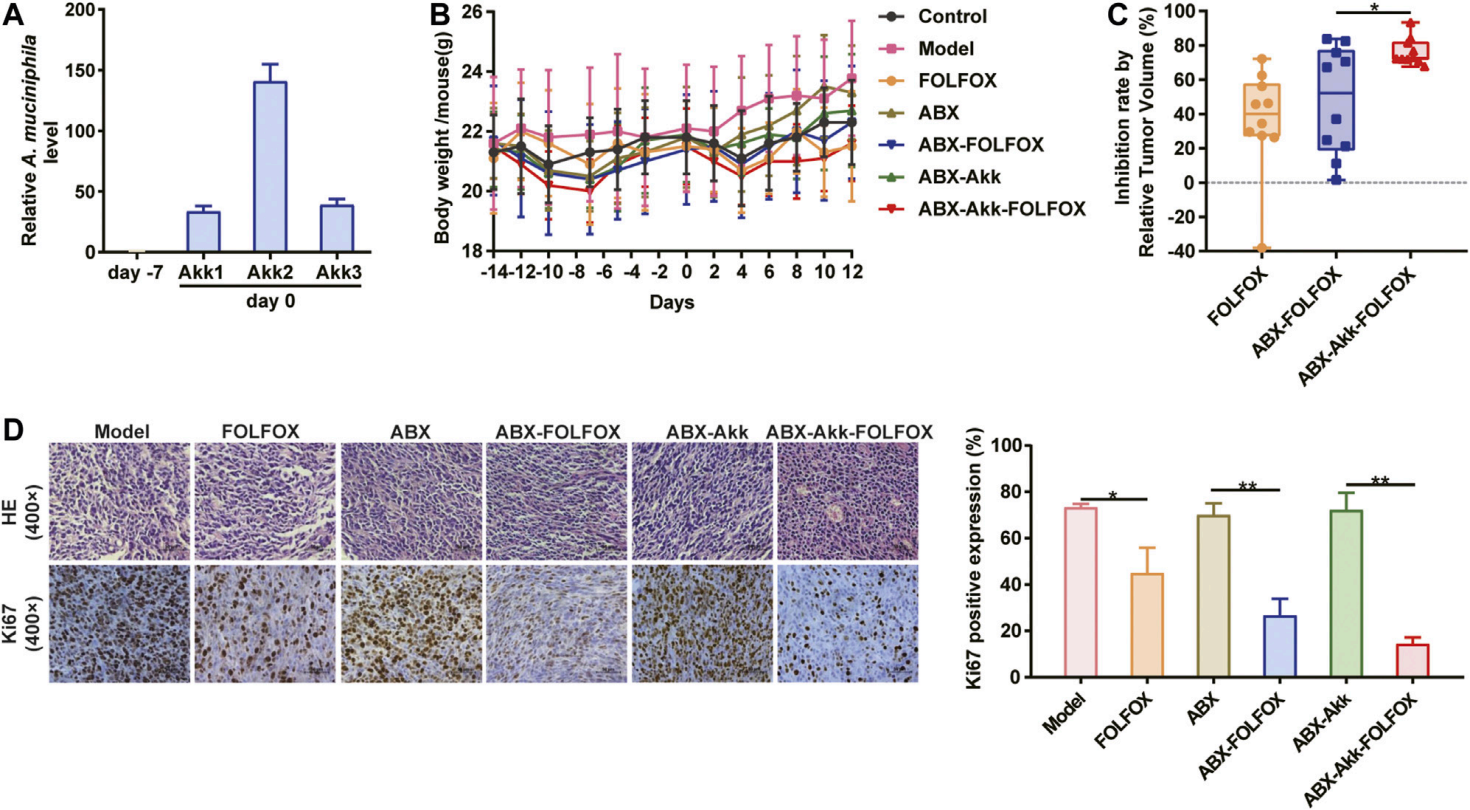
*A. muciniphila* colonization significantly enhanced the anti-cancer effect of FOLFOX. **(A)** Relative abundance of *A. muciniphila* before (day −7) and after (day 0) the transplantation. **(B)** Change of the body weight of tumor-bearing mice across the experiment. **(C)** Inhibition rate by RTV were calculated: Model *vs.* FOLFOX (36%), ABX *vs.* ABX-FOFOX (48%) and ABX-Akk *vs.* ABX-Akk-FOLFOX (76%). **(D)** The percentage of Ki67 positively stained cells. Data were presented as mean ± SD. The *p*-values < 0.05 were considered statistically significant, *p < 0.05, **p < 0.01.

To verify the influence of *A. muciniphila* on FOLFOX efficacy, *A. muciniphila* colonization combined with CT-26 colon cancer xenograft model was constructed (Supplementary Figure S6). The *A. muciniphila* transplantation was established referring to the published studies and our previous exploration (Gong et al., 2019). Three mice (labeled with Akk1, Akk2, Akk3) were randomly selected to evaluate the bacterial transplantation efficiency. As is shown in Figure 4A, the relative abundance of *A. muciniphila* remarkably increased after the colonization, indicating the success of model construction. First of all, FOLFOX treatment caused decreased body weight of tumor bearing mice, while *A. muciniphila* colonization did not influence it (Figure 4B). After pharmacodynamic evaluation, we found that the anti-cancer effect of FOLFOX was increased from 36 to 48% by ABX pretreatment, suggesting the involvement of gut microbiota in FOLFOX efficacy (Figure 4C). More importantly, the inhibition rate of FOLFOX was significantly enhanced (from 48 to 76%) with *A. muciniphila* colonization (*p* < 0.05) (Figure 4C). In addition, immunohistochemistry of Ki67 in tumor tissues further supported the above conclusion (Figure 4D). To summarize, our study confirmed that *A. muciniphila* transplantation could improve the efficacy of FOLFOX on colon cancer.

### Metabolomics Analysis Revealed Potential Gut Microbiota-Metabolite Axis Responsible for FOLFOX Efficacy

**FIGURE 5:**
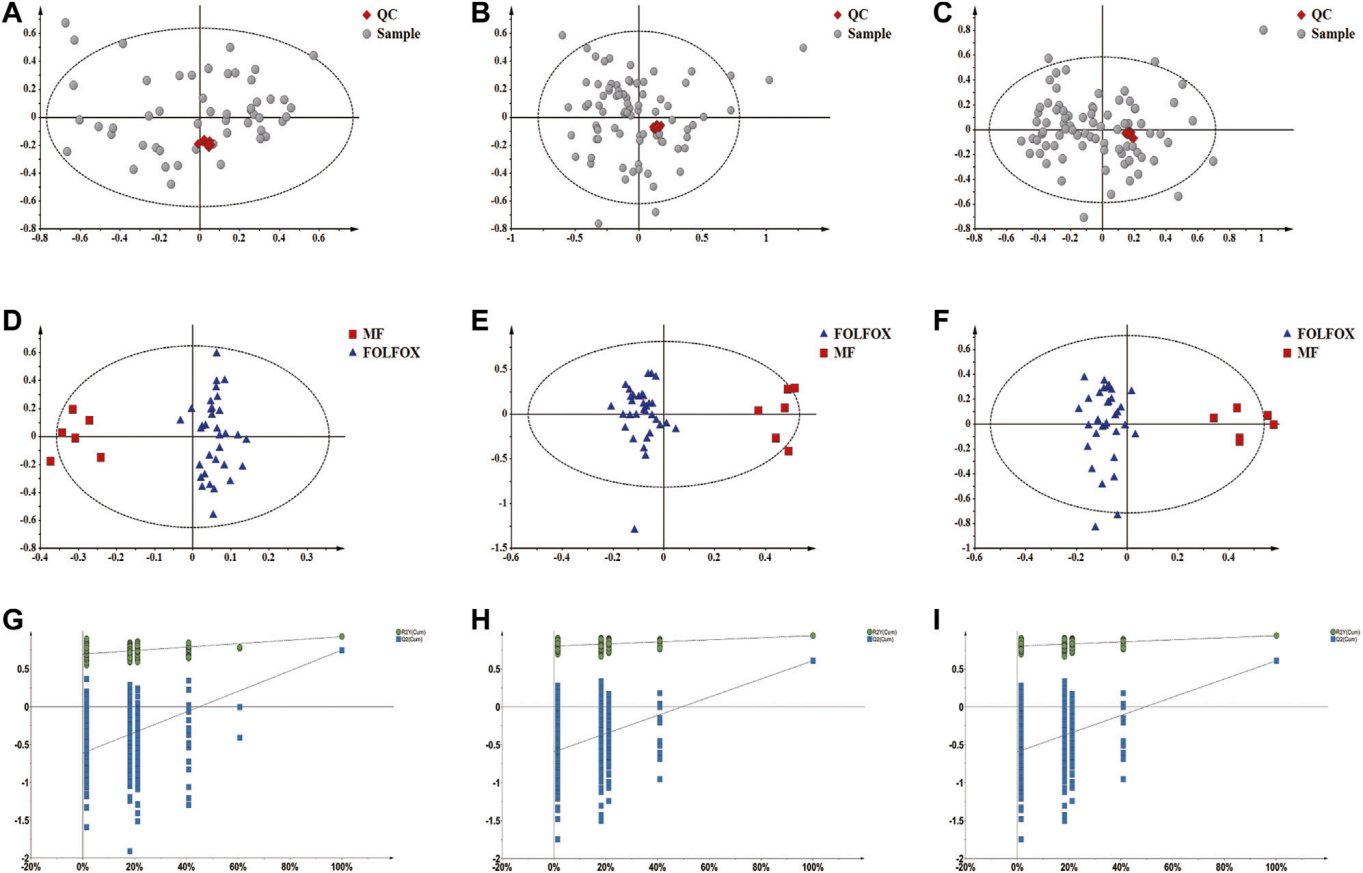
Non-target metabolomics analysis of fecal samples from the MF and FOLFOX groups. **(A–C)** QCs clustered very well in the PCA score plots constructed based on GC-MS, LC-MS (+) and LC-MS (−) data. **(D–F)** OPLS-DA score plots based on GC-MS (R^2^X: 0.474; R^2^Y: 0.929; Q^2^: 0.66), LC-MS (+) (R^2^X: 0.449; R^2^Y: 0.938; Q^2^: 0.607) and LC-MS (−) (R^2^X: 0.342; R^2^Y: 0.925; Q^2^: 0.746) data, respectively. **(G–I)** Permutation test result (500 times) of OPLS-DA models constructed from GC-MS, LC-MS (+) and LC-MS (−) data, respectively.

**FIGURE 6:**
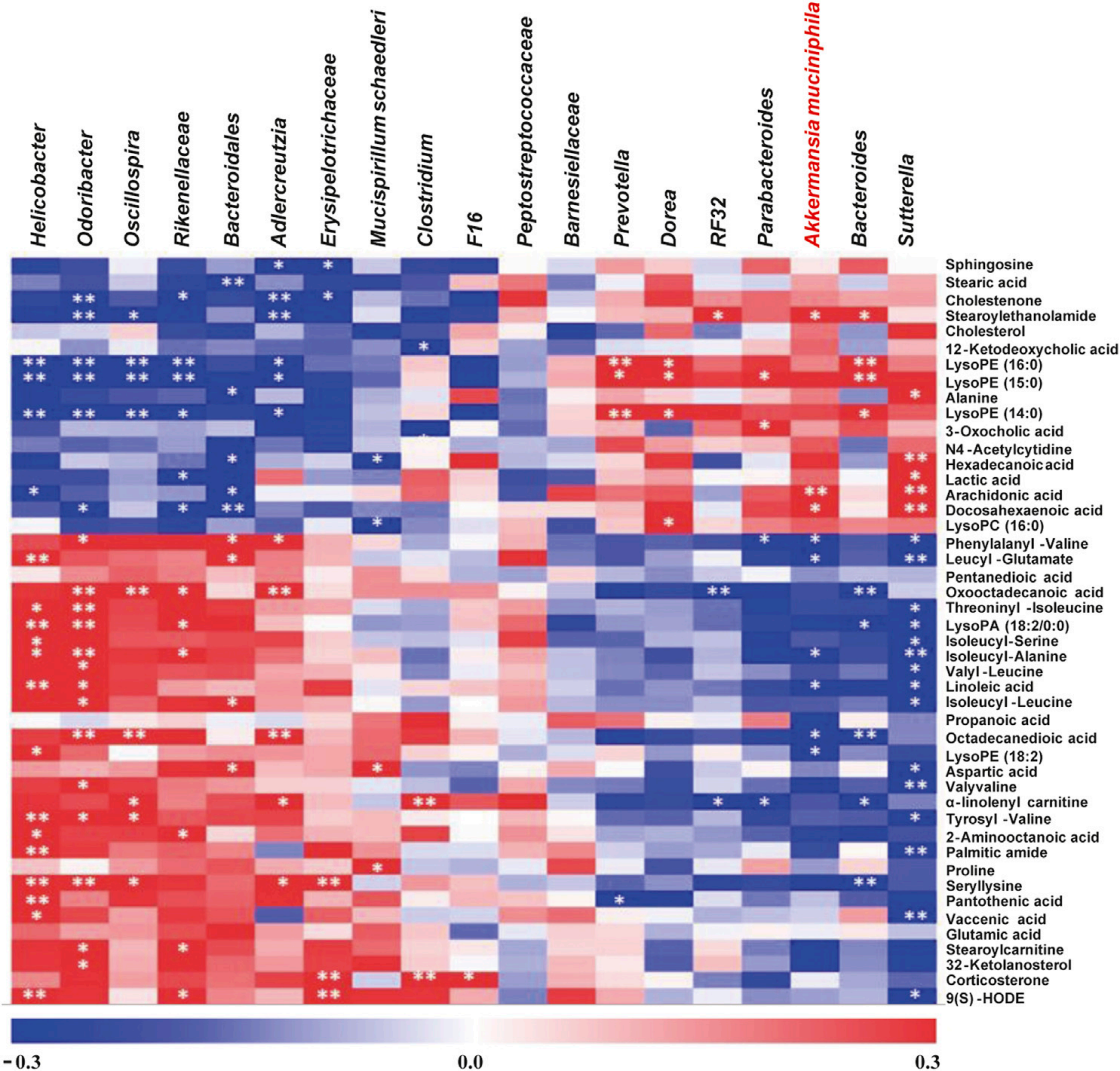
Non-target metabolomics analysis to explore the potential gut microbiota-metabolite axis for FOLFOX efficacy. The intensity of the colors represents the degree of association between the relative level of metabolites and the abundance of changed bacterial genera by Spearman’s correlations. The *p*-values < 0.05 were considered statistically significant, *p < 0.05, **p < 0.01.

It was well known that gut microbiota derived metabolites are important functional readouts of the gut microbiome and play essential roles in the action of chemotherapeutic drugs (Zhang et al., 2017b; Jia et al., 2018). Therefore, fecal samples from MF and FOLFOX groups were further subjected for non-target metabolomics analysis referring to our previous studies (Zhang et al., 2017a; Gao et al., 2019). In metabolomics, LC-MS is the most commonly applied platform. However, GC-MS with chemical derivatization is advantageous in acquiring polar metabolites such as carbohydrates and organic acids that are usually not well retained on a reverse phase column in LC-MS (Beale et al., 2018). Therefore, both GC-MS and LC-MS were utilized in the current study to achieve a wide coverage of metabolites. A tight clustering of the QCs in the PCA score plots was observed (Figures 5A–C), indicating good reproducibility of the methods. OPLS-DA models based on GC-MS and LC-MS were established to identify differential features between the two groups (Figures 5D–F). Permutation tests with 500 iterations were performed to confirm the OPLS-DA models were not overfitting (Figures 5G–I). As a result, 45 significantly changed metabolites were annotated with VIP > 1 and *p* < 0.05 (Supplementary Table S2). Spearman correlation analysis was performed to correlate the abundance of differential bacteria and annotated metabolites. As is shown in Figure 6, the relative level of stearoylethanolamide, arachidonic acid, and docosahexaenoic acid was positively correlated with the abundance of *A. muciniphila*, whereas phenylalanyl-valine, leucyl-glutamate, isoleucyl-alanine, linoleic acid, octadecanedioic acid, and lysoPE (18:2) had negative correlations with *A. muciniphila* (*p* < 0.05). Interestingly, we observed three branched-chain amino acid (BCAA) containing dipeptides (i.e., phenylalanyl-valine, leucyl-glutamate, isoleucyl-alanine) which might be potentially important mediators involving in the superior efficacy of FOLFOX.

## Discussion

Although oxaliplatin is extensively applied for colon cancer therapy, it was often combined with other chemotherapeutics because of the severe adverse effects and poor prognosis (Kang et al., 2020). In this study, oxaliplatin treatment exhibited a less satisfied anti-cancer effect and decreased body weight compared with FOLFOX, which is consistent with previous reports (Panebianco et al., 2018; Heshiki et al., 2020). FOLFOX is a regimen based on oxaliplatin in combination with 5-FU and calcium folinate, but the mechanism of its high efficacy and low toxicity is still unclear. Meanwhile, a limited response rate of FOLFOX (about 30–50%) existed in clinical practice (Wiseman et al., 1999), which is also confirmed in our study indicated by significantly individualized pharmacodynamic results. Importantly, emerging studies proposed “Pharmacomicrobiomics is the Holy Grail to Variability in Drug Response” (Sharma et al., 2019). Therefore, through the pharmacomicrobiomics approach, the study eventually focused on *A. muciniphila* for further verification.

*A. muciniphila* was firstly isolated from a sample of healthy human feces by Muriel Derrien in 2004, which is recognized as “beneficial bacteria” for its negative correlation with various diseases (cancer, diabetes, inflammatory bowel disease, autism, etc) (Ottman et al., 2017; Naito et al., 2018; Zhai et al., 2019; Zhang et al., 2019). As previously reported, *A. muciniphila* supplementation could restore the sensitivity of PD-1 inhibitor resistant individuals (Routy et al., 2018). Meanwhile, Chen *et al* also emphasized that *A. muciniphila* significantly improved the anti-tumor effect of cisplatin in Lewis lung cancer mice through immune-regulation (Chen Z. et al., 2020). In the current study, significantly increased efficacy of FOLFOX was observed when combined with *A. muciniphila* transplantation, which confirmed the importance of *A. muciniphila* for FOLFOX response. In brief, our findings may provide new insights into colon cancer therapy.

On the other hand, gut microbiota derived metabolites are important reflections of the distribution and function of the gut microbiome, which play pivotal roles in the interactions between the host and gut microbe (Sharma et al., 2019). In this study, nine metabolites were focused for their significant correlations with the abundance of *A. muciniphila*. Notably, three of them are BCAA containing dipeptides (i.e., phenylalanyl-valine, leucyl-glutamate, and isoleucyl-alanine). Recently, researchers have characterized the increased levels of dipeptides in various cancers (Wu et al., 2013; Li et al., 2019; Ozawa et al., 2020; Stolzenberg-Solomon et al., 2020). More importantly, Li *et al* recruited 3,482 participants for metabolomics analysis, and BCAA contained dipeptide glutamine-leucine was eventually confirmed as potential metabolic markers for early-stage colorectal cancer (Li et al., 2019). Thus, the negative correlations between *A. muciniphila* and these dipeptides observed in our study may reveal the possible gut microbiota-metabolites axis for gut bacteria mediated FOLFOX efficacy. Nevertheless, the relationship between *A. muciniphila* and the dipeptides as well as the underlying signal pathways remain to be elucidated.

Our study suggested the potential role of *A. muciniphila* in FOLFOX response and revealed the possible gut microbiota-metabolites axis which might be responsible for mediating FOLFOX efficacy. However, there are some limitations. First of all, FOLFOX was intraperitoneally administrated to tumor bearing-mice and the period is only 12 days. An extended experimental duration simulating the clinical practice in which several cycles of FOLFOX is applied or analyzing samples from patients might strengthen the clinical guidance. Meanwhile, the effect of *A. muciniphila* on FOLFOX efficacy was confirmed by bacteria colonization in our study, whether a decreased abundance of *A. muciniphila* would have a negative impact needs to be verified. Moreover, whether *A. muciniphila* colonization could influence the efficacy of oxaliplatin as well, further experiments are required. In addition, while up to 19 bacterial genera were initially identified to associate with FOLFOX treatment, we only verified the function of *A. muciniphila* which was the most significantly shifted. Whether the rest were involved in the efficacy of FOLFOX requires further explorations.

In this study, pharmacomicrobiomics approach was applied to investigate the involvement of gut microbiota in the anti-cancer effect of FOLFOX. As a result, *A. muciniphila* was selected for functional verification based on the 16S rDNA gene sequencing and correlation analysis results. The bacterial colonization experiment demonstrated the key role of *A. muciniphila* in FOLFOX efficacy. Metabolomics analysis further revealed a gut microbiota-metabolite axis that might be responsible for FOLFOX efficacy. In a word, this study highlighted the importance of *A. muciniphila* for the therapeutic effect of FOLFOX, providing a novel and effective strategy for clinical colon cancer treatment.

## Data Availability

The original contributions presented in the study are publicly available. This data can be found here: National Center for Biotechnology Information (NCBI) BioProject database under accession number PRJNA706146.
