# Supplementary material for: Akkermansia Muciniphila Potentiates the Antitumor Efficacy of FOLFOX in Colon Cancer
Source: Front Pharmacol. 2021 Sep 17;12:725583. doi: 10.3389/fphar.2021.725583 (PMC8484791; doi:10.3389/fphar.2021.725583)
Supplement: Supplementary file 1 [file DataSheet1.pdf]

Supplementary Material

1. Supplementary Methods

Non-target metabolomics analysis

Sample preparation

For GC-MS analysis, about 20 mg fecal samples were homogenized with precooled saline (4 μL/mg). Methanol was added to the fecal homogenate (4:1, μL/μL), vortex mixed for 15 min and centrifuged twice (4 ℃, 12000 rpm, 10 min). Then, 80 μL of the supernatant was transferred into a brown glass vial and added with 25 μL methoxyamine hydrochloride (10 mg/mL in pyridine). The mixture was incubated at 37 ℃ for 90 min and vacuum dried at 50 ℃ for 2 h (Labconco CentriVap® , Kansas, USA). Lastly, 120 μL of MSTFA was added to the dried extracts and incubated at 37 ℃ for 2 h, the supernatant was collected for GC-MS analysis.

For LC-MS analysis, about 20 mg fecal samples were homogenized with precooled saline (4 μL/mg). Methanol was added to the fecal homogenate (4:1, μL/μL), vortex mixed for 15 min and centrifuged twice (4 ℃, 12000 rpm, 10 min). The supernatant was transferred to LC vials for instrument analysis.

Instrument parameters

GC-MS. Column temperature was started at 70 ℃ (0 to 2 min), followed by an increase from 70 to 320 ℃ at the rate of 10 ℃/min (2 to 27 min) and maintained at 320 ℃ (27 to 29 min). The flow rate of carrier gas (Helium) was 1.0 mL/min. Injection volume was 1μL and the split ratio was 30:1. The temperatures of the injector, transfer line and ion source was 250, 250, and 200 ℃, respectively. Electron impact mode (EI, 70 eV) using full scan mode (from m/z 45 to 600) was carried out for data acquisition.

LC-MS. The column temperature was 40 ℃. The mobile phase consists of (A) 0.1% formic acid in water and (B) acetonitrile. The elution program (flow rate: 0.4 mL/min) was as follows: mobile phase A was decreased from 95 to 5% from 0 to 20 min, and maintained at 5% A for 3 min, then brought back to 95% A and maintained from 23 to

30 min. Curved desorption line (CDL) and heat block temperature were both 200 ℃. Electrospray ionization (ESI) source in both positive and negative modes with full scan mode (from m/z 100 to 1000) was carried out. Injection volume was 5μL. TOF analyzer detector voltage was 1.80 kV. The interface voltage was 4.5 kV for positive ion mode and - 3.5 kV for negative ion mode.

Quality control and differential metabolites screening

To check the robustness of the non-target metabolomics workflow, quality control samples (QC) were prepared by pooling equal aliquot of each sample, processed together with actual samples, and injected every six samples in the analytical sequence. Orthogonal partial least-squares-discriminant analysis (OPLS-DA) models were constructed to explore the differences between model and treatment groups. The variable importance in projection (VIP) generated from OPLS-DA models and *p* values from non-parametric Mann–Whitney U test (SPSS 19.0, Chicago, USA) were used to determine whether a feature is significantly different between the two groups. Only the features meet the requirements of VIP > 1 and $p < 0.05$ were considered for metabolite annotation.

Metabolite annotation

Identification of the metabolite detected by GC-MS was performed by comparing the mass spectra with those available in National Institute of Standards and Technology (NIST 11) library (with similarity ≥ 80%), and they were further confirmed by retention time, accurate mass and mass spectra with available standard compounds in the laboratory.

For LC-MS analysis, compound formula was firstly predicted based on theoretical, observed m/z values and isotopic patterns performed by Formula Predictor in LC/MS Solution software. Then, the m/z values, formulae and the MS/MS fragmentations were compared with online databases, such as HMDB (http://www.hmdb.ca), METLIN Metabolite (http://metlin.scripps.edu) and the Mass Bank (http://www.massbank.jp), etc. The identified metabolites were further confirmed by retention time, accurate mass and mass spectra with available standard compounds.

2. Supplementary Figures

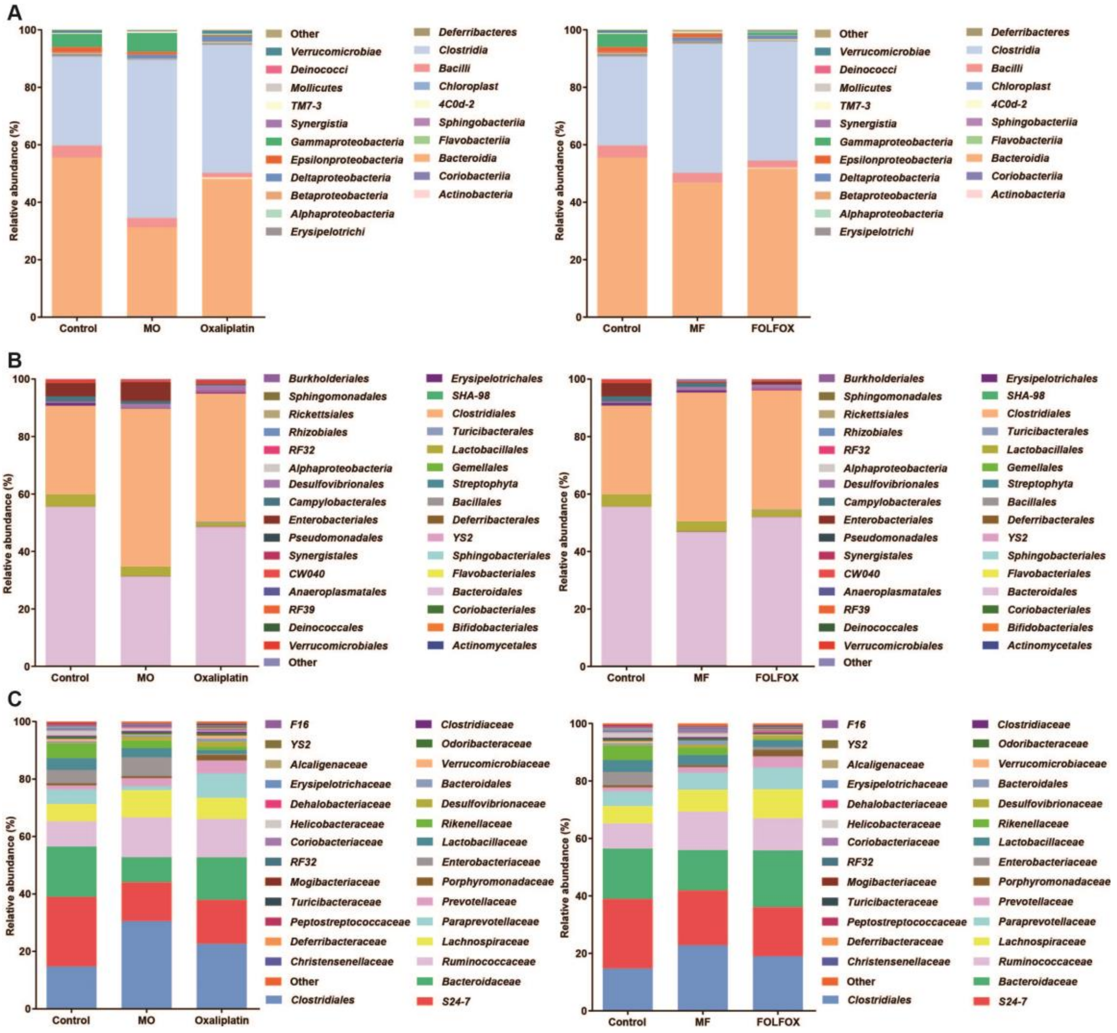

Figure S1 Relative bacterial distribution at (A) Class, (B) Order and (C) Family levels according to fecal 16S rDNA sequencing analysis.

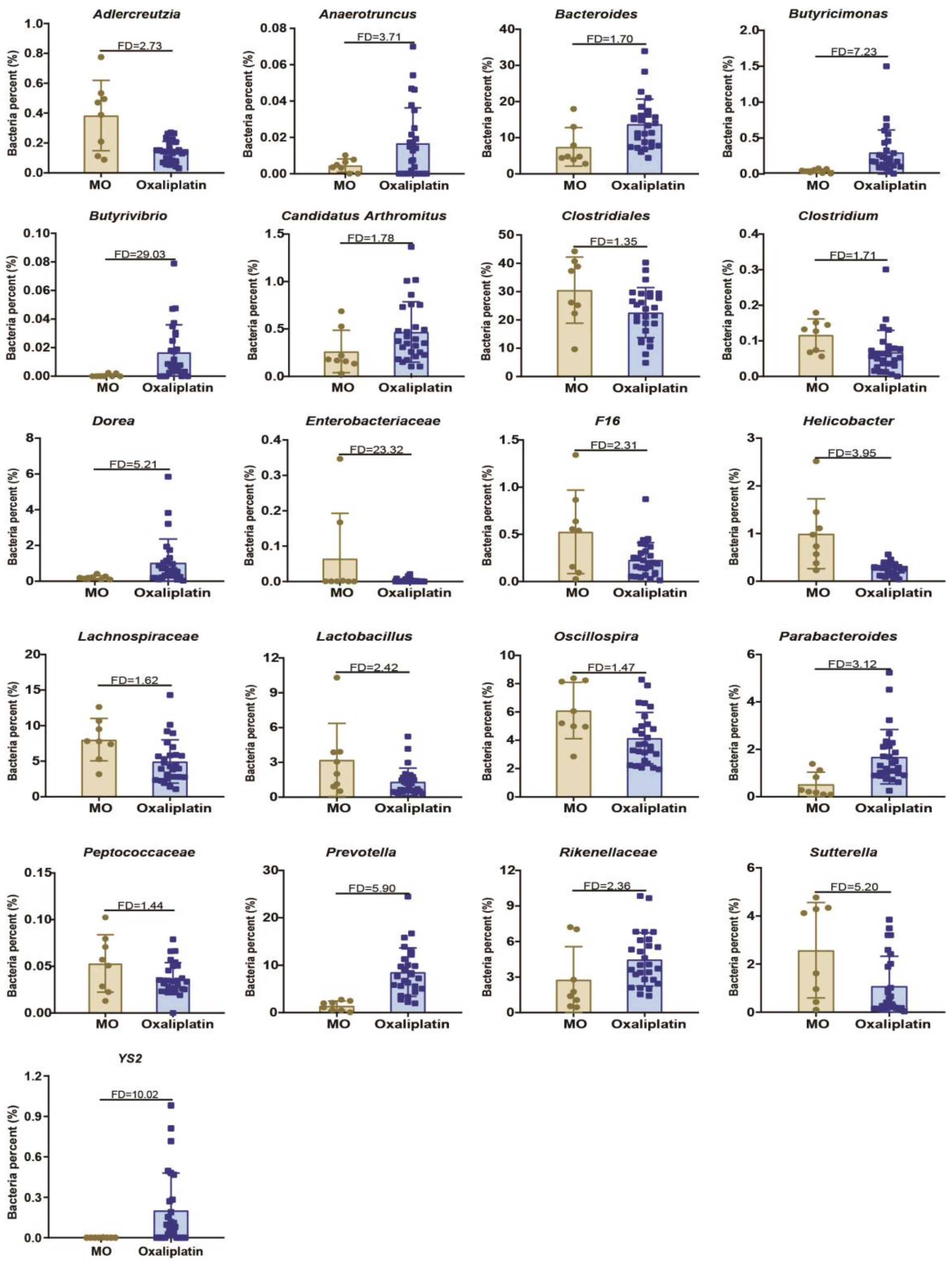

Figure S2 Relative abundance of significantly changed bacterial genera between MO and Oxaliplatin group (FD: Fold Change).

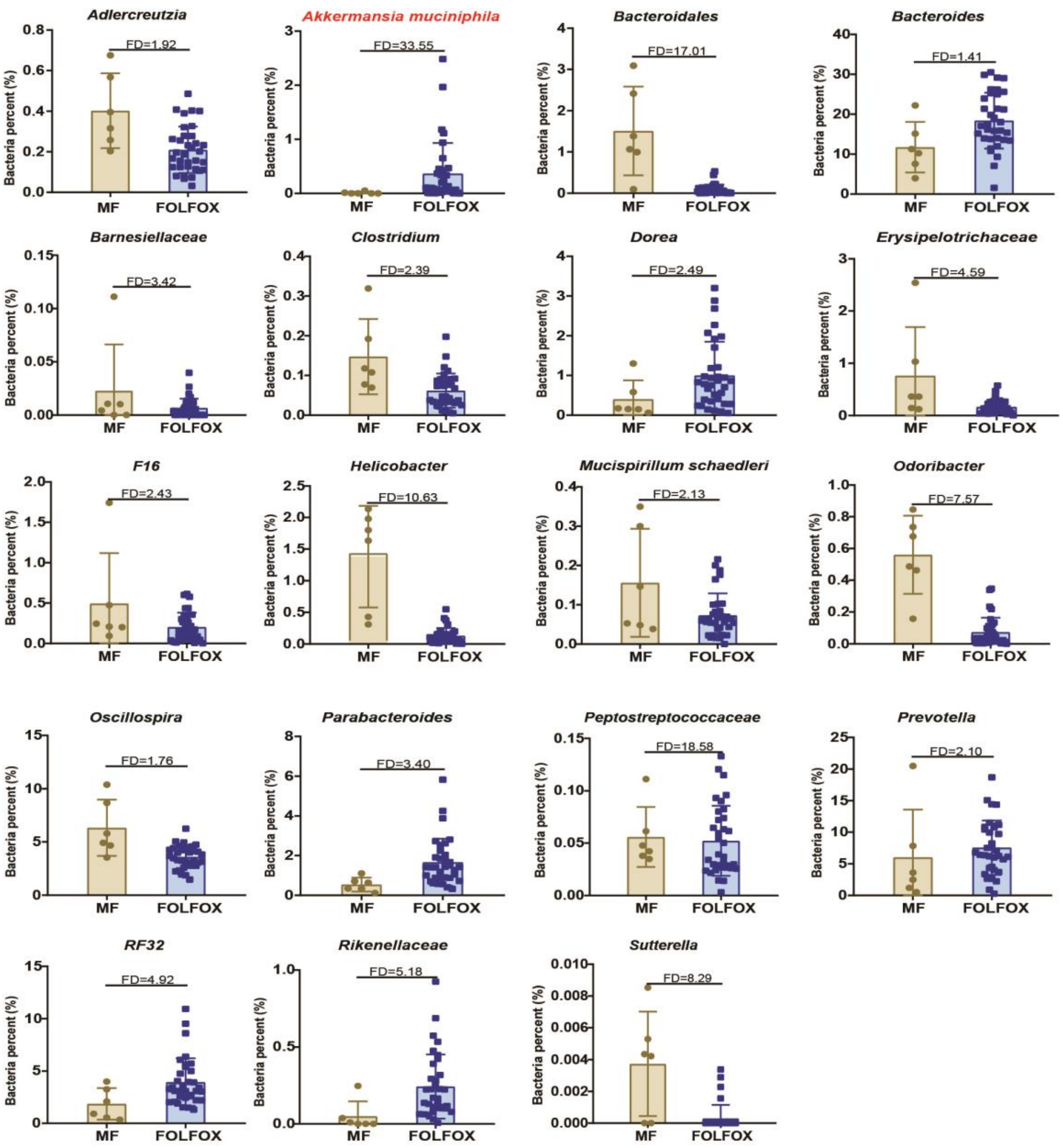

Figure S3 Relative abundance of significantly changed bacterial genera between MF and FOLFOX group (FD: Fold Change).

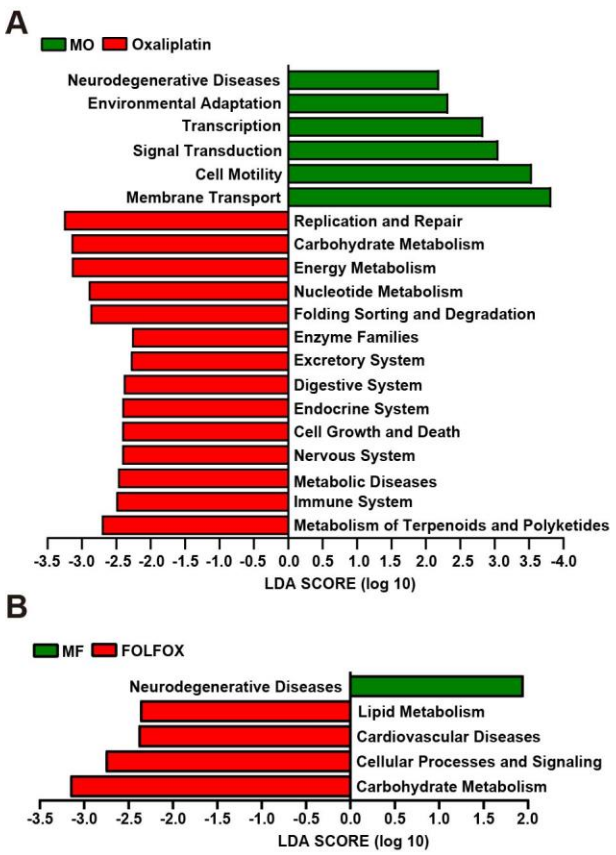

Figure S4 Predictive LDA scores of the differentially abundant KEGG pathways between (A) oxaliplatin *vs.* MO and (B) FOLFOX *vs.* MF (Wilcoxon rank-sum tests, $p < 0.05$).

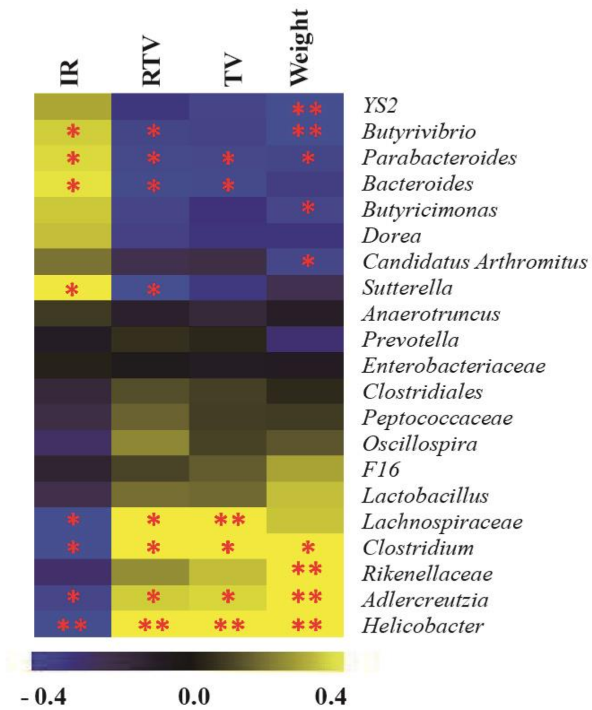

Figure S5 Heatmap of Spearman correlation coefficient between pharmacodynamic indices and abundance of changed bacterial genera after oxaliplatin treatment. The intensity of the colors represents the degree of association between the level of pharmacodynamic indices and abundance of changed bacterial genera measured by Spearman's correlations. IR: Inhibition rate calculated by Relative Tumor Volume. The p-values<0.05 were considered statistically significant, *$p<0.05$, **$p<0.01$.

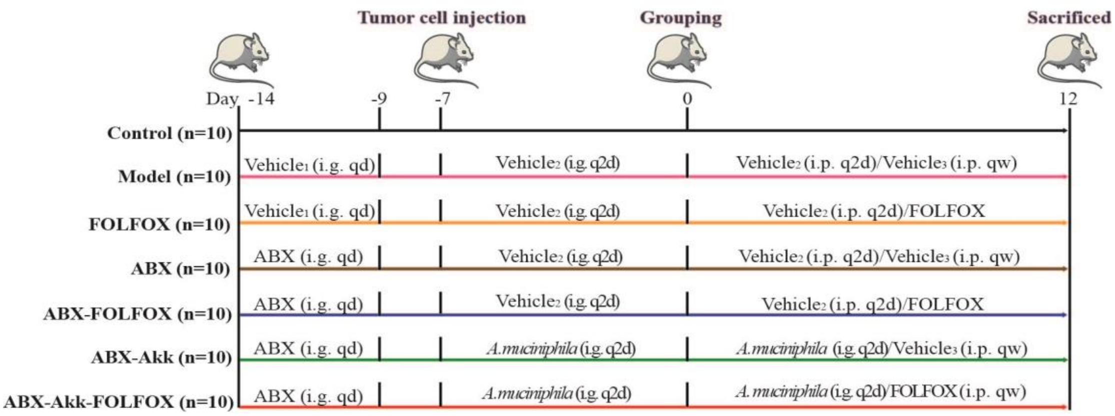

Figure S6 Schematic diagram of the experiment investigating *A. muciniphila* transplantation on FOLFOX efficacy. ABX: 100 mg/kg Vancomycin, 200 mg/kg Neomycin sulfate, 200 mg/kg Metronidazole and 200 mg/kg Ampicillin; FOLFOX (6 mg/kg oxaliplatin, 2 hours after 50 mg/kg 5-FU and 90 mg/kg Calcium Folinate); $Vehicle_1$: Vehicles of ABX; $Vehicle_2$: Vehicles of *A. muciniphila*; $Vehicle_3$: Vehicles of FOLFOX; qd (once a day); q2d (every other day); qw (once a week).

3. Supplementary Tables

Table S1. PCR Primers

Primer	Direction	Sequence (5’ → 3’)
Total bacteria detection	Forward	GTGSTGCAYGGYTGTCGTCA
	Reverse	ACGTCRTCCMCACCTTCCTC
A. muciniphila	Forward	CGAGACTGCCCAGATCAACT
	Reverse	TGCGGTTGGCTTCAGATACT

Table S2. Differential metabolites in fecal samples between MF and FOLFOX group.

**No.**	Metabolites	VIP	*p* value	Fold change (FOXFOL/MF)
1	Lactic acid	1.88	0.047	↑2.45
2	Alanine	11.48	0.004	↑1.48
3	Propanoic acid	2.62	0.029	↓1.37
4	Aspartic acid	1.46	0.009	↓1.57
5	Proline	1.81	0.024	↓1.53
6	Pentanedioic acid	2.50	0.047	↓2.75
7	Glutamic acid	1.13	0.029	↓1.51
8	Pantothenic acid	1.18	0.039	↓1.50
9	Linoleic acid	1.12	0.014	↓1.55
10	Stearic acid	1.64	0.039	↑2.05
11	Arachidonic acid	1.38	0.043	↑2.04
12	Docosahexaenoic acid	1.73	0.001	↑12.23
13	Hexadecanoic acid	8.51	0.002	↑1.65
14	Cholesterol	1.03	0.011	↑1.52
15	2-Aminooctanoic acid	1.77	0.002	↓2.06
16	Valyvaline	1.03	0.039	↓2.09
17	Isoleucyl-Serine	1.20	0.018	↓2.05
18	Threoninyl-Isoleucine	1.13	0.018	↓2.93
19	Isoleucyl-alanine	1.18	0.01	↓2.56
20	Tyrosyl-Valine	1.04	0.003	↓3.22
21	Leucyl-Glutamate	1.36	0.002	↓2.49
22	Valyl-Leucine	1.26	0.036	↓2.34
23	Isoleucyl-Leucine	1.61	0.011	↓2.65
24	Phenylalanyl-Valine	1.49	0.004	↓3.12
25	LysoPA(18:2/0:0)	1.71	0.011	↓2.67
26	N4-Acetylcytidine	1.27	0.006	↑1.42
27	3-Oxocholic acid	1.13	0.009	↑2.66
28	Corticosterone	2.00	0.004	↓2.34
29	12-Ketodeoxycholic acid	1.42	0.016	↑2.56
30	Octadecanedioic acid	2.29	0.006	↓2.85
31	LysoPE(14:0)	1.20	0.013	↑2.00
32	LysoPE(15:0)	2.45	0.004	↑2.55
33	LysoPE(18:2)	1.14	0.009	↓2.71
34	Sphingosine	1.23	0.043	↑1.38

35	LysoPE(16:0)	2.28	0.022	↑1.59
36	α-linolenyl carnitine	1.38	0.004	↓2.05
37	LysoPC(16:0)	1.50	0.036	↑1.78
38	Stearoylethanolamide	1.62	0.001	↑2.67
39	Oxooctadecanoic acid	1.50	0.022	↓1.86
40	9(S)-HODE	1.08	0.006	↓1.89
41	Palmitic amide	1.51	0.006	↓2.21
42	Vaccenic acid	1.72	0.047	↓1.73
43	Stearoylcarnitine	1.35	0.01	↓2.62
44	32-Ketolanosterol	2.17	0.013	↓2.28
45	Cholestenone	1.08	0.001	↑1.73
